# Fucoidan Ameliorates Oxidative Stress, Inflammation, DNA Damage, and Hepatorenal Injuries in Diabetic Rats Intoxicated with Aflatoxin B_1_

**DOI:** 10.1155/2020/9316751

**Published:** 2020-02-10

**Authors:** Mohammed S. Aleissa, Saad Alkahtani, Mabrouk Attia Abd Eldaim, Ali Meawad Ahmed, Simona G. Bungău, Bader Almutairi, May Bin-Jumah, Abdullah A. AlKahtane, Mohamed S. Alyousif, Mohamed M. Abdel-Daim

**Affiliations:** ^1^Department of Biology, Science College, Al Imam Mohammad Ibn Saud Islamic University (IMSIU), Riyadh, Saudi Arabia; ^2^Department of Zoology, College of Science, King Saud University, P.O. Box 2455, Riyadh 11451, Saudi Arabia; ^3^Department of Biochemistry and Chemistry of Nutrition, Faculty of Veterinary Medicine, Menoufia University, 32511, Egypt; ^4^Department of Food Hygiene, Faculty of Veterinary Medicine, Suez Canal University, 41522 Ismailia, Egypt; ^5^Department of Pharmacy, Faculty of Medicine and Pharmacy, University of Oradea, Oradea, Romania; ^6^Biology Department, College of Science, Princess Nourah Bint Abdulrahman University, Riyadh, Saudi Arabia; ^7^Pharmacology Department, Faculty of Veterinary Medicine, Suez Canal University, Ismailia 41522, Egypt

## Abstract

The current study was carried out to evaluate the ameliorative effect of fucoidan against aflatoxicosis-induced hepatorenal toxicity in streptozotocin-induced diabetic rats. Sixty-four Wister albino male rats were randomly assigned into eight groups (8 rats each) that received normal saline, fucoidan (FUC) at 100 mg/kg/day orally for 4 weeks, streptozotocin (STZ) at 50 mg/kg/i.p. single dose, STZ plus FUC, aflatoxin B_1_ (AFB_1_) at 50 *μ*g/kg/i.p. after one month of the beginning of the experiment for 2 weeks, AFB_1_ plus FUC, STZ plus AFB_1_, or STZ plus AFB_1_ and FUC. Injection of rats with STZ induced hyperglycemia. Rats with STZ-induced diabetes, with or without AFB_1_ intoxication, had significantly elevated activities of serum aspartate aminotransferase, alanine aminotransferase, and alkaline phosphatase, and levels of serum urea, creatinine, cholesterol, 8-oxo-2′-deoxyguanosine, interleukin-1*β*, interleukin-6, and tumor necrosis factor-*α*. In addition, these rats exhibited increased lipid peroxidation and reduced glutathione concentration and activities of superoxide dismutase, catalase, and glutathione peroxidase enzymes in the hepatic and renal tissues. In contrast, administration of FUC to diabetic rats, with or without AFB_1_ intoxication, ameliorated the altered serum parameters, reduced oxidative stress, DNA damage, and inflammatory biomarkers, and enhanced the antioxidant defense system in the hepatic and renal tissues. These results indicated that FUC ameliorated diabetes and AFB_1_-induced hepatorenal injuries through alleviating oxidative stress, DNA damage, and inflammation.

## 1. Introduction

Diabetes mellitus (DM) is a leading cause of morbidity and mortality worldwide. In developing countries, DM ranks as the 5^th^ most common cause of death [1]. Diabetes mellitus is classified into two types: insulin-dependent (that results from destruction of pancreatic *β* cells of Langerhans) and noninsulin-dependent (that results from defects in insulin action and/or secretion) [2]. Extensive research has shown that inflammation and oxidative stress are implicated in the development and complications of DM [3]. Streptozotocin (STZ) is used experimentally to induce DM in animals because it targets the *β* cells of Langerhans and induces permanent hyperglycemia in experimental animals [4].

Aflatoxins are produced by *Aspergillus flavus*, *Aspergillus parasiticus*, and *Aspergillus nominus* as secondary metabolites. Humans are exposed to aflatoxins through ingestion of contaminated food [5]. Storage of crops, such as corn and peanuts at excessive heat and humidity for long times, leads to proliferation of fungal spores and production of aflatoxins. The most prevalent and toxic aflatoxin is aflatoxin B_1_ (AFB_1_) [6]. Its toxic and carcinogenic activities are due to its bioactivation into AFB_1_ 8,9-epoxide by microsomal cytochrome P450. The resulting metabolite binds to DNA, RNA, and proteins, resulting in hepatic and renal damage [7]. Exposure of rats and pigs to AFB_1_ stimulates mRNA expression of tumor necrosis factor-*α* (TNF-*α*), interferon-*γ* (IFN-*γ*), and interleukin-6 (IL-6) [8]. The effects of AFB_1_ exposure depend on the dose and duration of treatment [6]. To the best of our knowledge, studies concerning the effects of mycotoxins on DM subjects are still rare. Although the liver plays vital roles in carbohydrate metabolism and regulation of blood glucose level, it is the target organ for AFB_1_ [9]. Intoxication of T1DM mice with AFB_1_-disordered T1DM elevated energy-producing mechanisms, gluconeogenesis, lipid, and oxidative phosphorylation, reduced major urinary protein 1, insulin sensitivity indicator, and subsequently elevated blood glucose level [10]. There is a positive interaction between AFB_1_ and diabetes in human subjects [11]. In addition, ochratoxin A induces toxic effects on the pancreatic tissue in a rat [12].

Fucoidans (FUCs) are highly sulfated polysaccharides, isolated from the cell walls of various species of brown seaweeds, such as *Saccharina japonica*, *Undaria pinnatifida*, and *Sargassum hemiphyllum*, and some animal species as sea cucumber [13]. *In vitro* and *in vivo* studies showed that FUCs have various biological activities such as hypoglycemic, nephroprotective, antioxidant, anti-inflammatory, anticoagulant, and antiviral effects [14, 15]. Many strategies are used to inhibit the development and progression of DM which rely on alleviating oxidative stress and inflammation [16]. The current study was aimed to evaluate the ameliorative potential of FUC against aflatoxicosis-induced hepatorenal toxicity in streptozotocin-induced DM in rats.

## 2. Materials and Methods

### 2.1. Chemicals

Streptozotocin and aflatoxin B_1_ were purchased from Sigma Chemical Co. (St. Louis, MO, USA). Fucoidan (Laminaria Japonica, as 500 mg/capsule) was obtained from Absunutrix Lyfetrition (USA). The kits, used for determination of blood glucose and serum metabolites levels, were obtained from BioDiagnostics Co. (Cairo, Egypt). ELISA kits, used to measure the serum levels of inflammatory cytokines, were obtained from R&D (Mannheim, Germany), while the kits for 8-OhdG measurement were purchased from Cayman Chemical (Co., MI, USA).

### 2.2. Animals

Sixty-four Wister albino male rats of 180 to 200 g weights were bought from the Egyptian Organization for Biological Products and Vaccines. Rats were kept at 25 ± 2°C and 12 h light/dark cycle in a well-ventilated room. Rats were given an access to food and water *ad libitum*. Rats were maintained under these environmental conditions for one week for adaption before the beginning of the experiment. The experimental design was approved by the Research Ethical Committee of the Faculty of Veterinary Medicine, Suez Canal University, Ismailia, Egypt (Approval No. 201616).

### 2.3. Experimental Design

Rats were randomly assigned into eight different experimental groups (8 rats each).

*The control* rats were given normal physiological saline.

*The second group* rats were given FUC at 100 mg/kg/day orally [17] between weeks 5 and 8 of the experiment.

*The third group* rats were administered STZ at 50 mg/kg/i.p. (dissolved in 0.1 mmol/l citrate buffer, pH 4.5) after 12 h fasting at the beginning of the experiment [18].

*The fourth group* rats were administered STZ as the third group and FUC as the second group.

*The fifth group* rats were given AFB_1_ at 50 *μ*g/kg/i.p. during the fifth and sixth weeks [19].

*The sixth group* rats were administered AFB_1_ as the fifth group and FUC as the second group.

*The seventh group* rats were administered STZ as the third group and AFB_1_ as the fifth group.

*The eighth group* rats were administered STZ as the third group, AFB_1_ as the fifth group, and FUC as the second group.

**Figure 1:**
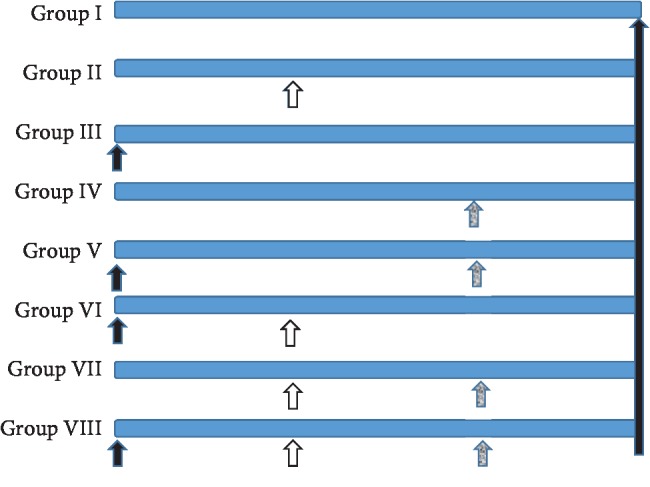
Design and animal allocation into different experimental treatments. White arrow indicates the start of FUC treatment. Black arrow indicates the administration of streptozotocin dose, and the grey arrow indicates the start of aflatoxin B_1_ treatment.

The experiment design is illustrated in Figure 1.

### 2.4. Blood and Tissue Sampling

Blood samples were collected at the end of the experiment. Blood samples were left to clot at room temperature for 30 min and then centrifuged at 2500 rpm for 15 min, and sera samples were separated and stored at -20°C till biochemical assessment. The rats were later sacrificed by decapitation and the liver and kidney tissues were collected and washed with normal physiological saline solution. Then, tissue samples were homogenized in ice-cold buffer containing 50 mM sodium phosphate-buffered saline (100 mM Na_2_HPO_4_/NaH_2_PO_4_) (pH 7.4), containing 0.1 mM EDTA then centrifuged for 30 minutes at 5000 rpm. The supernatant was collected and maintained at -80°C for subsequent analysis.

### 2.5. Biochemical Assays

The initial and fasting blood glucose levels were colorimetrically assayed according to Trinder [20] The activities of serum alanine aminotransferase (ALT) and aspartate aminotransferase (AST) were analyzed according to Reitman and Frankel [21]. The activity of serum alkaline phosphatase (ALP) was evaluated according to Tietz et al. [22].

The levels of serum total cholesterol according to (Richmond, 1973; Allain et al. 1974), urea, and creatinine were evaluated spectrophotometrically (Coulombe and Favreau [23] and Larsen [24]), respectively.

The tissue homogenates were used to determine the concentrations of malondialdehyde (MDA) [25], nitric oxide (NO) [26], reduced glutathione (GSH) [27], superoxide dismutase (SOD) [28], glutathione peroxidase (GSH-Px) [29], and catalase (CAT) [30] activities in both hepatic and renal tissues according to the referenced methods.

### 2.6. Evaluation of DNA Oxidation Biomarker

The concentration of serum 8-oxo-2′-deoxyguanosine (8-OhdG) was determined by using 8-OhdG competitive assay kit (Cayman Chemical Co., MI, USA) that detects free 8-OHdG and DNA-pound 8-OhdG.

### 2.7. Determination of Inflammatory Biomarkers

The serum levels of IL-1*β*, IL-6, and TNF-*α* were determined, by here using commercially available ELISA kits obtained from R&D (Mannheim, Germany) according to the manufacturers' instructions.

### 2.8. Statistical Analysis

All data were expressed as the means ± SEM, using SPSS software (version 20 for Windows, Armonk, NY). Data were analyzed by here using one-way ANOVA followed by Duncan's post hoc test to test the significant differences between experimental groups. The differences among groups were considered statistically significant at *P* ≤ 0.05.

## 3. Results

### 3.1. Fucoidan Reduced STZ-Induced Hyperglycemia in Rats

Intraperitoneal STZ administration was associated with significant increases in initial and fasting blood glucose levels compared with control rats. However, treatments of diabetic rats (with or without AFB_1_ intoxication) with FUC (4^th^ and 8^th^ groups) significantly decreased blood glucose levels compared to diabetic, nontreated rats (3^th^ and 7^th^ groups). On the other hand, both AFB_1_ and FUC (2^nd^, 5^th^, and 6^th^ groups) had no significant effects on fasting blood glucose levels (Table 1).

### 3.2. Fucoidan Normalized AFB_1_-Induced Alterations in Serum Liver Function Biomarkers in Diabetic Rats

Treatment of rats with STZ and/or AFB_1_ (3^rd^, 5^th^, and 7^th^ groups) was associated with significant increases in serum activities of ALT, AST, and ALP (that was most prominent in the combination group). In contrast, treatment of diabetic rats with or without AFB_1_ intoxication with FUC (4^th^, 6^th^, and 8^th^ groups) normalized the activities of serum AST, ALT, and ALP. FUC alone had no significant effect on the activities of serum AST, ALT, and ALP compared to control rats (Table 1).

### 3.3. Fucoidan Ameliorated AFB-1-Induced Alteration in Serum Kidney Function Biomarkers in Diabetic Rats

Treatment of rats with STZ and/or AFB_1_ (3^rd^, 5^th^, and 7^th^ groups) was associated with significant increases in serum urea and creatinine levels (that was most prominent in the combination group). However, treatment of diabetic rats with or without AFB_1_ intoxication with FUC (4^th^, 6^th^, and 8^th^ groups) significantly reduced serum urea and creatinine levels, compared with the 5^th^ and 7^th^ groups. Treatment with FUC alone was not associated with significant changes in serum urea and creatinine levels compared with the control rats (Table 1).

### 3.4. Fucoidan Normalized AFB-1-Induced Alteration in Serum Cholesterol Levels in Diabetic Rats

Administration of STZ and/or AFB_1_ (3^rd^, 5^th^, and 7^th^ groups) was associated with significantly increased serum cholesterol levels in comparison to control rats. However, treatment of diabetic or nondiabetic rats intoxicated with AFB_1_, with FUC (4^th^, 6^th^, and 8^th^ groups), normalized serum cholesterol levels, compared with the 3^rd^, 5^th^, and 7^th^ groups. Treatment with FUC alone did not cause significant changes in serum cholesterol levels compared with the control rats (Table 1).

### 3.5. Fucoidan Normalized AFB-1-Induced Oxidative Stress in Rat Hepatic and Renal Tissues

Administration of rats with streptozotocin and/or AFB_1_ (3^rd^, 5^th^, and 7^th^ groups) was associated with significant increases in hepatic and renal tissue concentrations of MDA and NO in comparison to control rats. However, treatment of diabetic and nondiabetic rats intoxicated with AFB_1_ with FUC (4^th^, 6^th^, and 8^th^ groups) normalized MDA and NO concentrations in both hepatic and renal tissues (Tables 2 and 3).

In contrast, induction of diabetes and/or aflatoxin intoxication significantly reduced GSH concentrations and GSH-Px, SOD, and CAT activities in both hepatic and renal tissues in the 3^rd^, 5^th^, and 7^th^ groups in comparison to the control rats. Treatment of diabetic and nondiabetic rats intoxicated with AFB_1_ with FUC (4^th^, 6^th^, and 8^th^ groups) reversed the effects of both diabetes and AFB_1_ intoxication on the aforementioned parameters. Treatment with FUC alone significantly elevated GSH concentration and GSH-Px, SOD, and CAT activities in the hepatic and renal tissues compared with the control group (Tables 2 and 3).

### 3.6. Fucoidan Normalized AFB-1-Induced Elevation of Serum Levels of DNA Oxidation Biomarker and Inflammatory Cytokines

**Figure 2:**
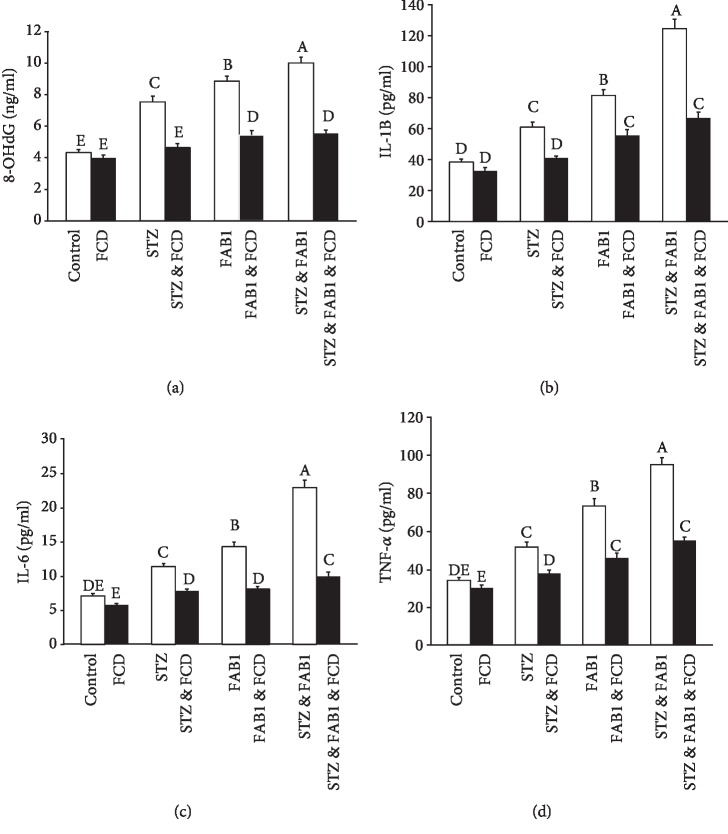
The ameliorative effect of FUC against AFB_1_-induced alteration in serum levels of 8-OHdG (a), IL-1*β* (b), IL-6 (c), and TNF-*α* (d) in streptozotocin-induced diabetic rats. Data are presented as the mean ± SEM. Columns having different letters are significantly different (*P* ≤ 0.05).

Induction of diabetes and/or AFB_1_ intoxication in the 3^rd^, 5^th^, and 7^th^ groups was associated with significantly elevated serum 8-OhdG, IL-1*β*, IL6, and TNF-*α* levels, compared to the control group. However, treatment of diabetic and nondiabetic rats intoxicated with AFB_1_ with FUC (4^th^, 6^th^, and 8^th^ group) reduced serum 8-OhdG, IL-1*β*, IL6, and TNF-*α* levels compared to nontreated rats (3^rd^, 5^th^, and 7^th^ groups). FUC itself had no significant effects on the serum 8-OhdG, IL-1*β*, IL6, and TNF-*α* levels in comparison to control rats (Figure 2).

## 4. Discussion

Great numbers of animals and people suffering from diabetes mellitus worldwide and its incidence increase in steady state and the number of diabetic patients has been expected to reach about 300 million in 2025 [31–33]. Numerous human and animals all over the world are subjected to mycotoxins because they frequently occur in food and feed stuffs [34]. The most prevalent and toxic aflatoxin worldwide is AFB_1_ [35–37]. Aflatoxin B_1_ induces several cellular damages through generation of free radicals and induction of lipid peroxidation resulting in oxidative stress in animals or humans. Oxidative stress plays indispensable role in AFB_1_-induced toxicity [38, 39] through activation of inflammatory cytokines such as TNF-*α*, IL-1*β*, and IL-6 [40]. Thus, exposure of diabetic patients to mycotoxicosis is unavoidable that is adversely affecting their health through induction of oxidative stress and subsequent inflammation.

The current study showed that injection of rats with STZ induced hyperglycemia, probably due to the irreversible cytotoxic effects of STZ on the *β* cells of the pancreas resulting in insulin deficiency [41]. Oxidative stress is implicated in this cytotoxic effect. In addition, induction of both diabetes and/or aflatoxicosis in rats resulted in elevated activities of liver function biomarkers, which can be explained by ROS generation, lipid peroxidation, and depleted antioxidant defense system in the hepatic tissue. These effects result in hepatocyte necrosis and release of hepatic enzymes into the circulation [42, 43]. Similarly, injection of rats with STZ and/or AFB_1_ significantly increased serum levels of urea and creatinine. These findings were in line with those of Eraslan et al. [44] and Zabad et al. [45]. This may be attributed to hyperglycemia and/or AFB_1_-induced ROS leading to necrosis of proximal tubular epithelial cells [44, 45]. To confirm the role of MD and AFB_1_-induced oxidative stress in disturbance of hepatorenal function, our study revealed that DM and/or AFB_1_ intoxication in rats induced oxidative stress in both hepatic and renal tissues as evidenced by the increased levels of MDA and NO and reduced concentration of GSH and activities of GSH-Px, SOD, and CAT (Tables 2 and 3). These results are in accordance with prior studies [46, 47]. In the presence of nitric oxide synthase, superoxide and NO react to generate peroxynitrite that injures the cell membrane and cellular biomolecules [48]. Further, these radicals attack the cellular DNA, as evidenced by the increased levels of serum 8-OHDG (Figure 1). In addition, AFB_1_ metabolites form AFB_1_–DNA adducts that induce DNA and cell damages and inhibit enzyme and protein synthesis through binding to nucleoproteins and nucleic acids [49]. Diabetes mellitus and AFB_1_ intoxication-induced oxidative stress in this study were associated with increased production of proinflammatory cytokines, IL-1*β*, IL-6, and TNF-*α* (Figure 1) leading to hepatorenal injuries and the elevation of activities and levels of their function biomarkers. There is extensive documentation in the literature of the association between oxidative stress and expression of proinflammatory cytokines such as IL-1*β*, IL-6, and TNF-*α*. In DM, glucose interacts with the amino groups of proteins producing advanced glycation end products that enhance the expression of some inflammatory and angiogenic cytokines [50]. A former study in pigs revealed that exposure to AFB_1_ enhances TNF-*α*, IFN-*γ*, and IL-6 expression [51]. In addition, AFB_1_ activates the expression of nuclear factor kappa B (NF*κ*B) and hence the production of inflammatory cytokines [52].

On the contrary, treatment of diabetic rats with FUC ameliorated the hepatorenal toxic effects of DM and/or AFB_1_ as evidenced by reduced blood glucose levels, activities of serum AST, ALT, and ALP, and serum levels of urea, creatinine, 8-OHDG, IL-1B, IL-6, and TNF-*α*. These findings were parallel with those of Wang et al. who concluded that FUC reduces STZ-induced hyperglycemia and kidney damage in rats [53]. The glucose-lowering effect of FUC might be due to enhancement of insulin secretion by pancreatic cells, increasing glucose uptake, or reduction of basal lipolysis [54]. Similarly, FUC improved the liver functions in carbon tetrachloride, microcystin, and diazinon-induced hepatorenal injuries in murine models [55–57] and lowered serum AST and ALT activities in hepatitis C virus-infected subjects [58]. These effects may be explained by FUC antioxidant activity as evidenced by alleviated lipid oxidation and enhancement of the antioxidant defense system in the liver and kidneys (Tables 2 and 3) and [14, 55]. These results were in accordance with previous published investigations. Fucoidans exert its antioxidant activity through scavenging ROS such as hydroxyl, peroxyl, and superoxide radicals [59, 60], and stimulating the activities of cellular SOD, CAT, GSH-Px, GST, and glucose-6-phosphate dehydrogenase [61]. In addition, our study showed that FUC reduces the production of proinflammatory cytokines (Figure 1). FUC has been shown to suppress the expression of NF*κ*B, protein kinase B, extracellular signal-regulated kinase, c-Jun N-terminal kinase, and p38 mitogen-activated protein kinase [62]. Moreover, it reduced LPS-induced elevation of serum levels of TNF-*α*, IL-1*β*, and IL-6 in mice [63]. Further, it alleviated aspirin-induced elevation of PGE2 and IL-6 plasma levels and increased the expression of IL-10 (anti-inflammatory cytokine) in rats [64]. Therefore, FUC ameliorated DM and AFB_1_-induced hepatorenal damages through suppressing oxidative stress-induced DNA damage and proinflammatory cytokine production.

In conclusion, DM and AFB_1_-induced hepatorenal injuries are probably mediated by oxidative stress, DNA damage, and inflammation. However, treatment with FUC ameliorated DM and AFB_1_-induced hepatorenal injuries, mostly due to its antioxidant and anti-inflammatory effects.

## Figures and Tables

**Table 1:** Effects of fucoidan on serum biochemical parameters of diabetic rats intoxicated with aflatoxin B_1_.

Parameters	Experimental groups							
	Control	FUC	STZ	STZ+FUC	AFB_1_	AFB_1_+FUC	STZ+AFB_1_	STZ+AFB_1_+FUC
*i* blood glucose (mg/dl)	83.37^b^ ± 3.50	84.21^b^ ± 3.75	281.29^a^ ± 5.80	274.42^a^ ± 5.82	91.19^b^ ± 4.12	82.36^b^ ± 2.59	285.54^a^ ± 6.42	276.22^a^ ± 6.39
*f* blood glucose (mg/dl)	89.53^c^ ± 2.1	81.24^c^ ± 3.68	305.12^a^ ± 8.14	140.91^b^ ± 5.82	87.88^c^ ± 3.62	90.68^c^ ± 5.00	322.45^a^ ± 7.95	128.88^b^ ± 5.40
AST (U/l)	26.56^d^ ± 0.32	24.49^d^ ± 1.19	70.28^c^ ± 4.13	31.7^d^ ± 1.36	84.67^b^ ± 4.42	34.33^d^ ± 2.41	149.73^a^ ± 5.82	33.08^d^ ± 2.08
ALT (U/l)	15.23^d^ ± 0.63	15.19^d^ ± 0.24	43.64^c^ ± 3.25	20.17^d^ ± 0.95	53.67^b^ ± 3.19	18.58^d^ ± 1.12	67.49^a^ ± 3.54	18.53^d^ ± 1.05
ALP (U/l)	28.90^d^ ± 1.40	26.13^d^ ± 0.84	77.56^c^ ± 2.50	34.05^d^ ± 1.47	87.33^b^ ± 3.63	32.09^d^ ± 2.23	105.97^a^ ± 4.41	32.43^d^ ± 2.34
Cholesterol (mg/dl)	91.76^d^ ± 4.51	88.07^d^ ± 3.91	189.10^b^ ± 5.08	111.64^d^ ± 481	158.62^c^ ± 4.45	91.86^d^ ± 4.43	215.87^a^ ± 6.57	101.45^d^ ± 4.79
Urea (mg/dl)	27.7^e^ ± 1.18	26.51^e^ ± 1.22	52.36^c^ ± 1.18	35.72^e^ ± 1.78	61.77^b^ ± 4.41	38.32^d^ ± 2.34	75.82^a^ ± 3.30	43.11^d^ ± 2.52
Creatinine (mg%)	0.33^d^ ± 0.05	0.30^d^ ± 0.07	1.21^c^ ± 0.05	0.54^d^ ± .04	3.16^b^ ± 0.28	0.77^d^ ± 0.04	4.72^a^ ± 0.36	0.92^d^ ± 0.05

Data are expressed as the means ± SEM (*n* = 8). *i* blood glucose: initial blood glucose; *f* blood glucose: fasting blood glucose; FUC: fucoidan; STZ: streptozotocin; AFB_1_: aflatoxin B_1_; ALT: alanine transferas; AST: aspartate transferase; ALP: alkaline phosphatase. Values having different superscripts within the same row are significantly different (*P* ≤ 0.05).

**Table 2:** Effects of fucoidan against aflatoxin-induced changes in diabetic rats' liver tissue oxidative stress and antioxidant biomarkers.

Parameters	Experimental groups							
	Control	FUC	STZ	STZ+FUC	AFB_1_	AFB_1_+FUC	STZ+AFB_1_	STZ+AFB_1_+FUC
MDA (nmol/g)	189.96^d^ ± 12.42	182.14^d^ ± 10.18	318.22^c^ ± 14.17	225.00^d^ ± 9.77	424.06^b^ ± 20.19	204.29^d^ ± 6.11	595.82^a^ ± 20.86	211.84^d^ ± 8.52
NO (*μ*mol/g)	106.51^d^ ± 5.54	87.54^d^ ± 3.27	161.89^c^ ± 6.38	110.95^d^ ± 2.80	200.48^b^ ± 6.69	117.93^d^ ± 6.12	275.73^a^ ± 11.13	125.12^d^ ± 2.93
GSH (mg/g)	189.63^b^ ± 8.35	225.83^a^ ± 7.08	118.98^d^ ± 6.62	181.58^b^ ± 6.32	108.79^d^ ± 5.13	176.84^b^ ± 11.07	95.17^d^ ± 4.51	159.16^c^ ± 6.32
GSH-Px (mol/g)	186.26^b^ ± 14.42	225.11^a^ ± 11.72	95.43^c^ ± 4.16	183.66^b^ ± 6.32	76.71^d^ ± 5.43	174.44^b^ ± 6.33	62.42^d^ ± 5.46	162.48^b^ ± 4.94
SOD (U/g)	30.63^b^ ± 1.17	36.90^a^ ± 1.56	13.28^c^ ± 1.32	30.43^b^ ± 2.62	9.59^d^ ± 0.48	28.94^b^ ± 1.43	5.78^d^ ± 0.87	27.69^b^ ± 1.93
CAT (U/g)	3.42^ab^ ± 0.20	3.97^a^ ± 0.18	1.79^c^ ± 0.14	3.18^b^ ± 0.16	1.13^d^ ± 0.09	2.99^b^ ± 0.14	1.01^d^ ± 0.08	3.18^b^ ± 0.16

Data are expressed as the means ± SEM (*n* = 8). FUC: fucoidan; STZ: streptozotocin; AFB_1_: aflatoxin B_1_; MDA: malondialdehyde; NO: nitric oxide; GSH: reduced glutathione; GSH-Px: glutathione peroxidase; SOD: superoxide dismutase; CAT: catalase. Values having different superscripts within the same row are significantly different (*P* ≤ 0.05).

**Table 3:** Effects of fucoidan against aflatoxin-induced changes in diabetic rats' renal tissue oxidative stress and antioxidant biomarkers.

Parameters	Experimental groups							
	Control	FUC	STZ	STZ+FUC	AFB_1_	AFB_1_+FUC	STZ+AFB_1_	STZ+AFB_1_+FUC
MDA (nmol/g)	68.72^e^ ± 3.04	62.30^e^ ± 3.04	149.21^c^ ± 4.19	76.72^e^ ± 2.52	197.69^b^ ± 13.47	87.93^e^ ± 3.04	287.65^a^ ± 13.51	108.39^d^ ± 2.85
NO (*μ*mol/g)	92.88^e^ ± 2.92	78.82^e^ ± 3.67	194.32^c^ ± 10.56	98.70^e^ ± 10.56	243.29^b^ ± 10.67	111.46^e^ ± 6.70	315.74^a^ ± 14.88	138.41^d^ ± 7.80
GSH (mg/g)	83.67^b^ ± 4.52	99.70^a^ ± 5.37	46.55^d^ ± 2.85	77.55^b^ ± 2.98	39.05^d^ ± 2.85	68.67^c^ ± 4.52	16.55^e^ ± 2.85	57.42^c^ ± 2.70
GSH-Px (mol/g)	50.22^b^ ± 3.60	61.43^a^ ± 3.35	21.43^c^ ± 4.16	46.91^b^ ± 3.60	18.93^c^ ± 1.58	43.00^b^ ± 2.98	14.84^c^ ± 0.85	40.34^b^ ± 2.27
SOD (U/g)	18.18^b^ ± 0.44	22.13^a^ ± 0.85	9.14^e^ ± 0.44	15.55^c^ ± 0.45	6.83^f^ ± 0.44	14.98^c^ ± 0.48	4.40^g^ ± 0.44	13.00^d^ ± 0.48
CAT (U/g)	2.05^b^ ± 0.24	2.69^a^ ± 0.30	0.71^e^ ± 0.05	1.79^b^ ± 0.11	0.65^e^ ± 0.05	1.43^c^ ± 0.09	0.49^e^ ± 0.07	1.03^d^ ± 0.09

Data are expressed as the means ± SEM (*n* = 8). FUC: fucoidan; STZ: streptozotocin; AFB_1_: aflatoxin B_1_; MDA: malondialdehyde; NO: nitric oxide; GSH: reduced glutathione; GSH-Px: glutathione peroxidase; SOD: superoxide dismutase; CAT: catalase. Values having different superscripts within the same row are significantly different (*P* ≤ 0.05).

## Data Availability

All data will be available when required.

## Abbreviations

MDA: Malondialdehyde
NO: Nitric oxide
CAT: Catalase
SOD: Superoxide dismutase
GSH-Px: Glutathione peroxidase
GSH: Reduced glutathione
GST: Glutathione transferase
ROS: Reactive oxygen species
8-OHdG: 8-Oxo-2′-deoxyguanosine
IL-1*β*: Interleukin 1 beta
IL-6: Interleukin 6
TNF-*α*: Tumor necrosis factor alpha
ALP: Alkaline phosphatase
AST: Aspartate aminotransferase
ALT: Alanine aminotransferase.
